# Depression, anxiety, and stress in Korean general population during the COVID-19 pandemic

**DOI:** 10.4178/epih.e2022018

**Published:** 2022-01-18

**Authors:** Hooyeon Lee, Dongwoo Choi, Jung Jae Lee

**Affiliations:** 1 Department of Preventive Medicine, College of Medicine, The Catholic University of Korea, Seoul, Korea; 2 Data Link & Operation Team, Cancer Data Center, National Cancer Control Institute, National Cancer Center, Goyang, Korea; 3 Department of Psychiatry, Dankook University Hospital, Cheonan, Korea; 4 Department of Psychiatry, College of Medicine, Dankook University, Cheonan, Korea; 5 Chungcheongnam-do Mental Health Welfare Center, Hongseong, Korea

**Keywords:** COVID-19, Pandemics, Depression, Anxiety, Stress, Marital status

## Abstract

**OBJECTIVES:**

The aim of this study was to investigate the prevalence and risk factors of poor mental health in the general Korean population during the coronavirus disease 2019 (COVID-19) pandemic.

**METHODS:**

This cross-sectional, population-based, online survey-based study was conducted from November 5 to 20, 2020 and included adults aged 20-49 years in Chungnam Province, Korea. A total of 549 adults were included.

**RESULTS:**

In total, 18.8% of the participants had symptoms of depression, 10.6% had symptoms of anxiety, and 5.1% had a high level of perceived stress during the COVID-19 pandemic. Higher levels of stress (odds ratio [OR], 3.13; 95% confidence interval [CI], 1.13 to 8.67), anxiety (OR, 2.33; 95% CI, 1.09 to 4.49), and depression (OR, 3.00; 95% CI, 1.64 to 5.50) were found among never married, widowed, divorced, and separated people than among married/cohabiting/partnered participants. Participants who felt increased stress at home during the COVID-19 outbreak reported more depression (OR, 2.45; 95% CI, 1.49 to 4.05) and anxiety (OR, 2.42; 95% CI, 1.31 to 4.50). Women had higher risks of anxiety (OR, 1.97; 95% CI, 1.09 to 3.58) and stress (OR, 6.40; 95% CI, 2.30 to 17.85) than men. Participants with the highest household income were less likely to report symptoms of stress than those with the lowest household income (OR, 0.24; 95% CI, 0.06 to 0.96).

**CONCLUSIONS:**

The participants in this study exhibited poor mental health index scores, suggesting that some people are at risk for mental health problems during the COVID-19 pandemic. Being married was independently and significantly associated with a lower likelihood of depression, anxiety, and stress.

## GRAPHICAL ABSTRACT

[Fig f1-epih-44-e2022018]

## INTRODUCTION

The coronavirus disease 2019 (COVID-19) outbreak began in December 2019 and quickly aroused global attention. The pathogen that causes COVID-19 is more contagious and spreads more rapidly than those involved in previous epidemics and pandemics, such as severe acute respiratory syndrome and Middle East respiratory syndrome [1]. The extent and timeliness of public health measures and COVID-19 containment efforts have differed among countries. Efforts have included closing international borders, restricting domestic travel, introducing mandatory self-isolation for at-risk and symptomatic individuals, social distancing, and wearing face masks in public areas [2]. Compulsory measures, such as containment, quarantine, community control, and business and school closures, have been implemented in Korea [3].

In the face of this large-scale infectious public health event and enormous disruptions to daily life, many people are under unprecedented pressure and experiencing severe psychological distress due to persistent fear, social isolation, and economic hardship [4]. The wearing of face masks, disruption of interpersonal interactions due to social distancing, and loss of income have also been reported to affect mental health [2]. Although actions are by public health bodies are necessary to reduce the spread of COVID-19, but can lead to isolation, loneliness, and increased stress and anxiety.

A growing body of evidence has demonstrated the clinical effects of the COVID-19 pandemic on mental health, including higher rates of depression (14.6-48.3%), anxiety (6.3-50.9%), and stress (8.1-81.9%) [2,5,6]. The prevalence of depressive symptoms in the United States was more than three-fold higher during the COVID19 pandemic than before [7]. Risk factors for mental distress during the COVID-19 pandemic include women gender, younger age, and the presence of chronic/psychiatric illnesses [5]. Other factors predicting mental distress included loneliness, divorced/widowed or single status, and lower household income.

People without family are at a particularly high risk of health problems and deteriorated mental health due to COVID-19 [8,9]. However, the family situation, especially the parental burden, is difficult for families due to home schooling, social isolation, and lockdown situations. Family stress is also associated with pandemicrelated stress, anxiety, and depression [8]. COVID-19 has widened gaps among married people, single people, those living with others, and those living in isolation [10]. Marital status is a predictor of health outcomes in Western populations. However, data from Asian cultures remain limited [11].

The prevalence of mental health symptoms and associated factors during the COVID-19 outbreak need to be determined to set appropriate priorities for public health policies and establish effective healthcare interventions in Korea. Thus, we investigated the prevalence and risk factors of mental health symptoms in the general population in Korea during the COVID-19 pandemic.

## MATERIALS AND METHODS

### Data and study population

Through a web-based cross-sectional survey targeting adults aged 20-49 years in Chungnam Province, Korea, we investigated mental health, experiences, and perceptions of the COVID-19 pandemic, and the psychosocial effects of COVID-19.

Conventional face-to-face interviews were not feasible during the pandemic. Therefore, we collected data from an online survey panel, which Embrain Public® recruited through random sampling of residential addresses throughout Korea at the end of October 2020. Participants answered the questionnaires anonymously online from November 5 to 20, 2020.

Participants were people who decided to enter studies for which they were eligible by signing up on a panel platform. A total of 5,182 people were invited to participate via the Internet, of whom 993 (19.2%) agreed to participate. After the exclusion of participants with missing variables, 549 adults aged 20-49 years constituted the final study population. All participants provided informed consent prior to completing the survey.

### Dependent variables: depression, anxiety, and perceived stress

The primary outcomes were depression, anxiety, and perceived stress. We used the Korean version of the Patient Health Questionnaire-9 (PHQ-9) to determine whether participants had depressive symptoms [12]. Its 9 items assess the frequency of depressive symptoms over the past 2 weeks on a 4-point Likert scale ranging from 0 (rarely or none of the time) to 3 (most or all of the time). The PHQ-9 score ranges from 0 points to 27 points, and higher scores indicate more severe depressive symptoms. In our study, a PHQ-9 score ≥ 10 indicated the presence of depression [13-15].

We used the Korean version of the Generalized Anxiety Disorder-7 (GAD-7) scale to assess anxiety symptoms [16]. Its 7 items assess the frequency of anxiety symptoms over the past 2 weeks on a 4-point Likert scale ranging from 0 (never) to 3 (nearly every day). The total GAD-7 score ranges from 0 to 21, with higher scores indicating more severe functional impairment due to anxiety. A total GAD score ≥ 10 points was taken to indicate the presence of anxiety symptoms [17].

The Perceived Stress Scale (PSS) is a self-report instrument consisting of 10 items that assess “how unpredictable, uncontrollable, and overloaded respondents find their lives.” Each of the items on the PSS-10 is rated on a 5-point Likert scale, ranging from 0 (never) to 4 (very often). The PSS-10 consists of 6 positively worded items (items 1, 2, 3, 6, 9, and 10) and 4 negatively worded items (items 4, 5, 7, and 8). The negatively worded items were re-coded during the analysis. Total scores range from 0-40, with higher scores indicating higher levels of perceived stress. A high level of perceived stress was defined as a PSS-10 score ≥ 27 [18]. We used the Korean version of the PSS-10 [19].

### Independent variables

The mental health-related impact of COVID-19 was measured by 4 items, including support from friends, support from family members, financial stress, and stress at home. Scores were compared between the COVID-19 period and pre-COVID-19 period (much increased, increased, same as before, decreased, and much decreased).

The socio-demographic variables included gender, age, marital status, education level, and household income level. Marital status was classified as married/cohabiting/partnered or unmarried/widowed/divorced/separated. Age was categorized into groups of 20-29 years, 30-39 years, and 40-49 years. We categorized education level as high school graduation or less, or college or more. Household income was categorized as < US$2,000/mo, US$2,000-4,999/mo, or ≥ US$5,000/mo.

### Statistical analysis

First, we investigated the general characteristics of the study population, the prevalence rates of depression, anxiety, and stress, and the psychological impact of COVID-19. Second, we analyzed the prevalence of depression, stress, and anxiety during COVID-19 according to the socio-demographic characteristics and negative psychosocial changes using the chi-square test. Third, the bivariate associations between the independent variables and the mental health variables were calculated using logistic regression, and the associations were reported as odds ratios (ORs) with 95% confidence intervals. Fourth, all independent variables were entered simultaneously into multivariate logistic regression models and the associations were reported as adjusted ORs. All statistical analyses were performed using SAS version 9.4 (SAS Institute Inc., Cary, NC, USA). A p-value < 0.05 was considered to indicate statistical significance.

### Ethics statement

This study was approved by the Institutional Review Board of Dankook University (DKU 2021-05-038).

## RESULTS

Table 1 describes the socio-demographic characteristics and mental health status of the 549 participants aged 20-49 years. A total of 74.9% of the participants had a university level of education or above and 50.3% were married. Among all study participants, 18.8% had depression (PHQ-9 score ≥ 10). Anxiety symptoms (GAD-7 ≥ 10) were reported by 10.6% of the respondents. Furthermore, 5.1% of the participants reported high perceived stress (PSS-10 score ≥ 27). Comparing the COVID-19 period to the pre-COVID-19 period, 21.5% and 9.7% of the respondents felt that support from their friends and family members had decreased when they were in need. Financial stress and stress at home increased during the COVID-19 pandemic for 48.6% and 30.4% of respondents, respectively.

Table 2 summarizes the prevalence of depression, anxiety, and stress by the socio-demographic characteristics and negative psychosocial changes. Women were more likely to have anxiety and high perceived stress than men. Age was negatively associated with depression. Married people had lower levels of depression, anxiety, and stress than those who were never married or whose marriages ended in divorce or widowhood. Anxiety symptoms were more severe in people with less education and a lower household income than those with higher education and income levels. Depression and anxiety were associated with negative psychosocial changes such as decreased support from friends or family members and increased financial stress or stress at home.

Table 3 shows the results from multivariate logistic regression analysis, performed to identify risk factors for poor mental health. Women had ORs for anxiety and stress of 1.97 and 6.40, respectively. Subjects aged 30-39 years had 2.96 times higher perceived stress levels than those aged 20-29 years. Married people had significantly lower rates of depression, anxiety, and stress. People in the highest income group had lower perceived stress levels. Stress at home during the COVID-19 pandemic was positively associated with depression and anxiety. No significant relationships were detected between mental health and support from friends or family members.

## DISCUSSION

This study revealed high prevalence rates of depression, anxiety, and stress in the general population of Korea aged 20-49 years. In total, 10.6% of the participants were likely to have generalized anxiety disorder, as indicated by scores of 10 or higher on the GAD-7, which was much higher than the proportion of 2.4% reported in a previous national survey based on lifetime prevalence [20]. The prevalence of depressive symptoms (PHQ-9 score ≥ 10) was much higher than the rates of 6.1-6.7% reported in previous studies analyzing population-based data [21,22]. Compared to previous reports conducted before COVID-19 in Korea, our data suggest increased rates of depression and anxiety symptoms, although this conclusion should be drawn cautiously because of the inherently different assessment methods and sampling strategies.

Women were more likely to have anxiety and high perceived stress than men. Although this difference was not statistically significant, this study showed that women had a 1.23-fold higher likelihood of depression than men. Men and women exhibited different mental health outcomes, as women were more likely to have high anxiety and perceived stress levels than men [5,22-24].

Married people were less likely to suffer from poor mental health. In our study, being married was significantly associated with a lower likelihood of depression, anxiety, and stress. A considerable amount of evidence indicates that married individuals enjoy better mental health than single individuals [25,26]. A study assessing psychosocial distress, conducted in the United States, Korea, France, and Hong Kong, suggested that being married is associated with better well-being than being single [27].

Single subjects are certainly more likely to feel the effects of loneliness and isolation than married couples, which may explain their consistently higher levels of stress, anxiety, and depression [5,27]. Loneliness has been amplified during the COVID-19 crisis, which has been characterized by social distancing [28]. Such social distancing, in association with limited social support and interpersonal communication, has worsened depression, fear, insomnia, and anxiety symptoms [20]. Married participants had 40% lower odds of developing anxiety during the COVID-19 lockdown than unmarried participants [29]. Our study showed the mental health benefits of marriage in an Asian population. Being married per se is not universally beneficial, but the satisfaction and support associated with such a relationship tend to be important [30]. The importance of the quality of the interaction with a spouse or partner should be investigated.

In our study population, 30.4% of participants reported that stress at home had increased since the COVID-19 pandemic. Increased stress at home after the pandemic was positively associated with depression and anxiety after controlling for socio-demographic variables and marital status. Previous studies showed that parents with children at home reported unique pressures, including worrying about their children’s physical and mental health, education, and the stress of looking after their children while continuing to work [29,31]. Actions such as social distancing, sheltering in place, restricted travel, and closures of key community resources are likely to dramatically increase the risk of family violence [32].

Poor economic status is a significant risk factor for developing symptoms of mental disorders, especially depressive symptoms during the pandemic period [5]. However, our study showed household income was not significantly associated with depression and anxiety during the COVID-19 pandemic. One possibility is that government interventions to support financial hardship might mitigate poor mental health during the pandemic [33]. We need further evaluation to investigate association between mental health and social support or government financial aid program.

The prevalence rates of mental health symptoms have been reported to be different among studies, which could be due to the use of different measurement scales, patterns of reporting, or cultural factors [5]. Regional differences in the psychological health of the general public during major disease outbreaks exist according to outbreak severity, the economy, government preparedness, the availability of medical supplies/facilities, and the effectiveness of dissemination of COVID-related information. The stage of the outbreak in a given region also affects the psychological responses of the public.

The COVID-19 pandemic has been a traumatic event. In addition, the policies created to prevent its spread have introduced new stressors and disrupted daily living for infected patients and the general population [7]. Studies regarding the psychological implications of the COVID-19 pandemic are being published in various countries and indicate moderate-to-high levels of distress [28]. Our study does not suggest long-term effects of the COVID-19 pandemic on mental health. However, the prevalence of moderate to severe depressive and anxiety symptoms was higher than reported in several previous studies conducted before the COVID-19 pandemic [21,34]. Follow-up studies may be needed to assess the long-term psychological impacts of the COVID-19 pandemic [5].

This study had several limitations. First, although the number of active internet users has grown, the online modality has a risk of selection bias. Nevertheless, online surveys have become an important tool for COVID-19 research when conventional survey methods are not feasible [35]. Second, the survey also used selfreported questionnaires to measure psychiatric symptoms and did not make clinical diagnoses. Response bias may also have been present, where anxious individuals stressed by the pandemic may have been more likely to participate [2]. This could have resulted in worse reported mental well-being outcomes compared to the actual situation. Third, as the study participants were residents of a province, it was not possible to analyze regional differences, which makes it hard to generalize our results to the entire population of Korea. Finally, as this study had a cross-sectional design, the ability to make causal inferences regarding the associations between perceptions of COVID-19 and the prevalence of mental health symptoms is limited. The possibility of residual confounding remains, as not all potential confounders were controlled for in the model.

Despite these limitations, our findings have implications for psychological interventions aimed at reducing psychological distress, and improving mental health and psychological resilience, during public health emergencies. The COVID-19 pandemic has been controlled in Korea without a nationwide lockdown or drastic social distancing policies, unlike Europe and the United States [36]. Population-based studies on mental health and COVID-19 should be established to provide evidence-based guidance on responding to future pandemics in Korea.

The participants in this study exhibited relatively poor mental health index scores during the COVID-19 outbreak. Implementing community-based strategies to promote resilience and support psychologically vulnerable individuals during the COVID-19 crisis is of fundamental importance. More assistance should be provided to vulnerable groups in society, in accordance with age, gender, and marital status.

## Figures and Tables

**Figure f1-epih-44-e2022018:**
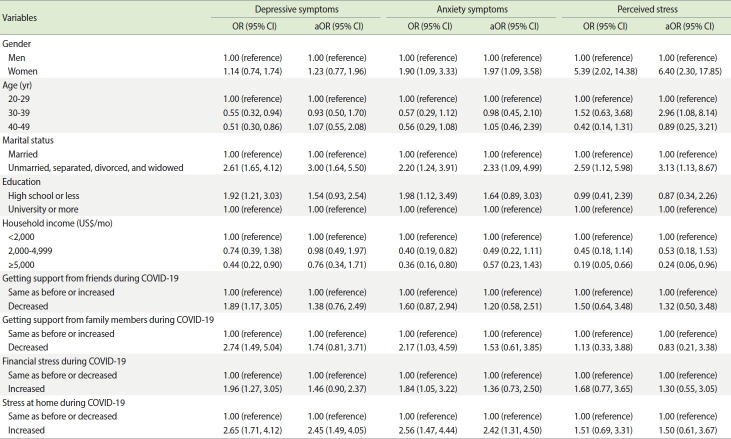


**Table 1.** Socio-demographic characteristics and mental health (n=549)

Characteristics	n (%)
Gender	
Men	286 (52.1)
Women	263 (47.9)
Age (yr)	
20-29	148 (27.0)
30-39	188 (34.2)
40-49	213 (38.8)
Marital status	
Married/cohabiting/partnered	276 (50.3)
Unmarried, separated, divorced, and widowed	273 (49.7)
Education	
High school or less	138 (25.1)
University or more	411 (74.9)
Household income (US$/mo)	
<2,000	62 (11.3)
2,000-4,999	376 (68.5)
≥5,000	173 (31.5)
Depressive symptoms (0-27)	
No	446 (81.2)
Yes (PHQ-9≥10)	103 (18.8)
Median [Q1-Q3]	4.0 [2.0-8.0]
Anxiety symptoms (0-21)	
No	491 (89.4)
Yes (GAD-7≥10)	58 (10.6)
Median [Q1-Q3]	3 [1.0-6.0]
Perceived stress (0-40)	
No	521 (94.9)
Yes (PSS-10≥27)	28 (5.1)
Median [Q1-Q3]	17 [14.0-21.0]
Support from friends during COVID-19	
Decreased	118 (21.5)
Same as before	375 (68.3)
Increased	56 (10.2)
Support from family members during COVID-19	
Decreased	53 (9.7)
Same as before	383 (69.8)
Increased	113 (20.6)
Financial stress during COVID-19	
Decreased	17 (3.1)
Same as before	265 (48.3)
Increased	267 (48.6)
Stress at home during COVID-19	
Decreased	39 (7.1)
Same as before	343 (62.5)
Increased	167 (30.4)

COVID-19, coronavirus disease 2019; PHQ-9, Patient Health Questionnaire-9; GAD-7, Generalized Anxiety Disorder-7.

**Table 2.** Mental health and psychosocial characteristics associated with coronavirus disease 2019 (COVID-19)

Variables		Depressive symptoms		Anxiety symptoms		Perceived stress	
		Yes (%)	p-value	Yes (%)	p-value	Yes (%)	p-value
Gender							
	Men	17.8	0.561	7.7	0.022	1.8	<0.001
	Women	19.8		13.7		8.8	
Age (yr)							
	20-29	26.4	0.021	14.9	0.138	5.4	0.037
	30-39	16.5		9.0		8.0	
	40-49	15.5		8.9		2.4	
Marital status							
	Married	11.7		7.0		2.9	
	Unmarried, separated, divorced, and widowed	25.7	<0.001	14.1	<0.001	7.3	0.021
Education							
	High school or less	26.8	0.005	15.9	0.018	5.1	0.986
	University or more	16.1		8.8		5.1	
Household income (US$/mo)							
	<2,000	25.8	0.051	21.0	0.017	11.3	0.021
	2,000-4,999	20.4		9.6		5.4	
	≥5,000	13.3		8.7		2.3	
Support from friends during COVID-19							
	Same as before or increased	16.5	0.009	9.5	0.125	4.6	0.349
	Decreased	27.1		14.4		6.8	
Support from family members during COVID-19							
	Same as before or increased	16.9	0.001	9.7	0.039	5.0	0.845
	Decreased	35.9		18.9		5.7	
Financial stress during COVID-19							
	Same as before or decreased	13.8	0.002	7.8	0.030	3.9	0.189
	Increased	24.0		13.5		6.4	
Stress at home during COVID-19							
	Same as before or decreased	13.9	<0.001	7.6	0.001	4.5	0.295
	Increased	29.9		17.4		6.6	

**Table 3.** Associations between mental health and sociodemographic and psychosocial characteristics associated with coronavirus disease 2019 (COVID-19)

Variables		Depressive symptoms		Anxiety symptoms		Perceived stress	
		OR (95% CI)	aOR (95% CI)	OR (95% CI)	aOR (95% CI)	OR (95% CI)	aOR (95% CI)
Gender							
	Men	1.00 (reference)	1.00 (reference)	1.00 (reference)	1.00 (reference)	1.00 (reference)	1.00 (reference)
	Women	1.14 (0.74, 1.74)	1.23 (0.77, 1.96)	1.90 (1.09, 3.33)	1.97 (1.09, 3.58)	5.39 (2.02, 14.38)	6.40 (2.30, 17.85)
Age (yr)							
	20-29	1.00 (reference)	1.00 (reference)	1.00 (reference)	1.00 (reference)	1.00 (reference)	1.00 (reference)
	30-39	0.55 (0.32, 0.94)	0.93 (0.50, 1.70)	0.57 (0.29, 1.12)	0.98 (0.45, 2.10)	1.52 (0.63, 3.68)	2.96 (1.08, 8.14)
	40-49	0.51 (0.30, 0.86)	1.07 (0.55, 2.08)	0.56 (0.29, 1.08)	1.05 (0.46, 2.39)	0.42 (0.14, 1.31)	0.89 (0.25, 3.21)
Marital status							
	Married	1.00 (reference)	1.00 (reference)	1.00 (reference)	1.00 (reference)	1.00 (reference)	1.00 (reference)
	Unmarried, separated, divorced, and widowed	2.61 (1.65, 4.12)	3.00 (1.64, 5.50)	2.20 (1.24, 3.91)	2.33 (1.09, 4.99)	2.59 (1.12, 5.98)	3.13 (1.13, 8.67)
Education							
	High school or less	1.92 (1.21, 3.03)	1.54 (0.93, 2.54)	1.98 (1.12, 3.49)	1.64 (0.89, 3.03)	0.99 (0.41, 2.39)	0.87 (0.34, 2.26)
	University or more	1.00 (reference)	1.00 (reference)	1.00 (reference)	1.00 (reference)	1.00 (reference)	1.00 (reference)
Household income (US$/mo)							
	<2,000	1.00 (reference)	1.00 (reference)	1.00 (reference)	1.00 (reference)	1.00 (reference)	1.00 (reference)
	2,000-4,999	0.74 (0.39, 1.38)	0.98 (0.49, 1.97)	0.40 (0.19, 0.82)	0.49 (0.22, 1.11)	0.45 (0.18, 1.14)	0.53 (0.18, 1.53)
	≥5,000	0.44 (0.22, 0.90)	0.76 (0.34, 1.71)	0.36 (0.16, 0.80)	0.57 (0.23, 1.43)	0.19 (0.05, 0.66)	0.24 (0.06, 0.96)
Getting support from friends during COVID-19							
	Same as before or increased	1.00 (reference)	1.00 (reference)	1.00 (reference)	1.00 (reference)	1.00 (reference)	1.00 (reference)
	Decreased	1.89 (1.17, 3.05)	1.38 (0.76, 2.49)	1.60 (0.87, 2.94)	1.20 (0.58, 2.51)	1.50 (0.64, 3.48)	1.32 (0.50, 3.48)
Getting support from family members during COVID-19							
	Same as before or increased	1.00 (reference)	1.00 (reference)	1.00 (reference)	1.00 (reference)	1.00 (reference)	1.00 (reference)
	Decreased	2.74 (1.49, 5.04)	1.74 (0.81, 3.71)	2.17 (1.03, 4.59)	1.53 (0.61, 3.85)	1.13 (0.33, 3.88)	0.83 (0.21, 3.38)
Financial stress during COVID-19							
	Same as before or decreased	1.00 (reference)	1.00 (reference)	1.00 (reference)	1.00 (reference)	1.00 (reference)	1.00 (reference)
	Increased	1.96 (1.27, 3.05)	1.46 (0.90, 2.37)	1.84 (1.05, 3.22)	1.36 (0.73, 2.50)	1.68 (0.77, 3.65)	1.30 (0.55, 3.05)
Stress at home during COVID-19							
	Same as before or decreased	1.00 (reference)	1.00 (reference)	1.00 (reference)	1.00 (reference)	1.00 (reference)	1.00 (reference)
	Increased	2.65 (1.71, 4.12)	2.45 (1.49, 4.05)	2.56 (1.47, 4.44)	2.42 (1.31, 4.50)	1.51 (0.69, 3.31)	1.50 (0.61, 3.67)

OR, odds ratio; aOR, adjusted odds ratio; CI, confidence interval.
